# Ibrutinib-Associated Liver Injury in a Patient with Chronic Lymphocytic Leukemia: Clinical Course and Therapeutic Approach

**DOI:** 10.3390/hematolrep17060069

**Published:** 2025-12-11

**Authors:** Antonio Frolli, Guido Parvis, Martina Bullo, Selene Grano, Giovanni Fornari, Valentina Bonuomo, Daniela Cilloni, Carmen Fava

**Affiliations:** 1 Department of Clinical and Biological Sciences, University of Turin, 10124 Turin, Italy; daniela.cilloni@unito.it; 2 Division of Hematology, AO Ordine Mauriziano di Torino, 10128 Turin, Italy

**Keywords:** ibrutinib, chronic lymphocytic leukemia, BTKi-related hepatotoxicity, ibrutinib-associated hepatotoxicity

## Abstract

**Background:** Ibrutinib, a Bruton’s tyrosine kinase inhibitor (BTKi), has revolutionized the treatment of Chronic Lymphocytic Leukemia (CLL), yet hepatotoxicity remains a rare and poorly characterized adverse event. **Case Presentation:** We report the case of a 54-year-old man with progressive CLL and radiologically confirmed hepatic involvement who developed acute hepatocellular injury during ibrutinib monotherapy. Baseline liver tests showed mild abnormalities attributed to hepatic steatosis and leukemic infiltration. After approximately 10–12 weeks of ibrutinib (420 mg/day), transaminases markedly increased (ALT 660 U/L, AST 326 U/L), while bilirubin and synthetic function remained normal. Viral, autoimmune, and obstructive causes were excluded. Stepwise dose reductions to 140 mg/day provided limited benefit. The addition of prednisone (50 mg/day) led to rapid biochemical improvement. Ibrutinib was successfully re-escalated to 280 mg/day alongside venetoclax initiation, maintaining disease control without recurrence of liver injury. **Discussion:** The temporal relationship, exclusion of alternative causes, and corticosteroid responsiveness suggest an ibrutinib-induced liver injury, possibly exacerbated by pre-existing hepatic steatosis and leukemic infiltration. **Conclusions:** This case underscores the multifactorial pathogenesis of BTKi-related hepatotoxicity and highlights the potential role of corticosteroids in management. Prompt recognition, stepwise dose adjustment, and corticosteroid therapy may enable continued treatment and sustained disease control in selected patients.

## 1. Introduction

Chronic Lymphocytic Leukemia (CLL) is the most common adult leukemia in Western countries and is characterized by the progressive accumulation of mature but functionally incompetent B lymphocytes [1]. Over the past decade, the management of CLL has undergone a paradigm shift with the advent of targeted therapies that inhibit key signaling pathways involved in B-cell proliferation and survival. Among these, Bruton’s tyrosine kinase inhibitors (BTKi) have become a cornerstone of treatment, replacing or complementing chemoimmunotherapy in most therapeutic settings [2].

Ibrutinib, the first-in-class BTK inhibitor, irreversibly binds to the cysteine-481 residue of BTK, thereby blocking B-cell receptor (BCR) signaling and downstream activation of NF-κB and other survival pathways [3]. Its efficacy was first demonstrated in randomized clinical trials, which established ibrutinib as a superior option compared to chemoimmunotherapy in CLL [4,5]. Subsequent trials confirmed its benefit in high-risk subgroups, such as patients harboring del(17p) or TP53 mutations, leading to its widespread approval as both monotherapy and in combination regimens [6].

More recently, time-limited combination therapies integrating BTKi and BCL-2 inhibitors have emerged as a promising strategy. The combination of ibrutinib with venetoclax has demonstrated deep and durable remissions, allowing treatment discontinuation while maintaining disease control [7,8]. Based on these data, the fixed-duration regimen of ibrutinib plus venetoclax has become an approved and guideline-endorsed option for previously untreated CLL [9].

Despite its well-documented efficacy, long-term tolerability remains a clinical challenge. With a median follow-up of approximately 17 months, real-world and trial-based analyses have shown that up to 41% of patients discontinued ibrutinib therapy, with a median time to discontinuation of about 7 months [10]. Importantly, drug-related adverse events are the leading cause of treatment cessation across all clinical settings. The most frequently reported toxicities include bleeding, atrial fibrillation, hypertension, diarrhea, rash, and infections. These toxicities are largely attributed to off-target effects, resulting from the inhibition of other kinases beyond BTK. Hepatotoxicity has been only rarely described with ibrutinib [7,8,9].

To date, only a few case reports and small series have documented liver injury related to BTKi therapy, and the underlying mechanisms remain poorly understood. Proposed hypotheses include idiosyncratic drug-induced liver injury (DILI), immune-mediated reactions, and hepatic involvement by CLL itself, which may complicate the diagnostic process [11,12,13,14]. Furthermore, the presence of hepatic infiltration by leukemic cells could predispose patients to a higher risk of liver dysfunction during targeted treatment, either by altering hepatic metabolism or by contributing to local inflammatory responses. Given the increasing use of BTK inhibitors in both frontline and relapsed settings, awareness of potential hepatic complications is essential for timely recognition and appropriate management. Here, we describe the case of a patient with CLL and hepatic involvement who developed acute liver toxicity during ibrutinib therapy. We detail the diagnostic work-up, therapeutic decisions, and clinical outcome, contributing to the limited but growing body of evidence on BTKi-associated hepatotoxicity.

## 2. Case Presentation

A 54-year-old man with a history of hypertension, obesity (Body Mass Index 31 kg/m^2^), and chronic iron-deficiency anemia (endoscopic work-up negative) was diagnosed with CLL in March 2024 based on peripheral blood immunophenotyping and histology on palatine tonsils; without other evidence of disease in other organs. At diagnosis, the disease harbored unmutated IGHV, wild-type TP53, no del(17p) and a biallelic del(13q) (82%). He was initially managed with active surveillance.

Restaging in March 2025 showed disease progression with widespread lymphadenopathy above and below the diaphragm and new hepatic involvement. Whole-body MRI demonstrated increased nodal burden and multiple ill-defined, confluent hepatic nodules with restricted diffusion, suspicious for leukemic infiltration; the spleen measured 15 cm. PET/CT revealed only faint metabolic activity in enlarged nodes (maximum SUV ~2.1), and target abdominal MRI with contrast medium confirmed a markedly steatotic liver with multifocal lesions compatible with hematologic infiltration, without biliary dilation or vascular involvement. Baseline laboratory testing before treatment initiation (April 2025) showed mild hepatic abnormalities: total bilirubin 1.42 mg/dL (direct 0.47, indirect 0.95), AST 54 U/L, ALT 92 U/L, and GGT 88 U/L; renal function and coagulation were normal; WBC 27.7 × 10^9^/L HB 16 g/dL and platelets 168 × 10^9^/L.

In April 2025, he started a fixed-duration ibrutinib–venetoclax therapy: ibrutinib 420 mg once daily, with venetoclax planned after 12 weeks. Ibrutinib was initially well tolerated.

After three months, liver enzymes rose to AST 92 U/L, ALT 147 U/L, and GGT 107 U/L; ibrutinib 420 mg was continued in light of the modest transaminase (grade 2) elevation e for the initial hepatic involvement. On July 2025, transaminases acutely worsened (grade 3—ALT 430 U/L, AST 224 U/L, GGT 150 U/L; LDH 329 U/L), prompting dose reduction of ibrutinib to 280 mg daily and discontinuation of concurrently prescribed acyclovir and allopurinol. Four days later, liver tests remained elevated (ALT 424 U/L, AST 179 U/L, GGT 226 U/L) with preserved synthetic function (normal PT/aPTT, albumin and bilirubin). Abdominal ultrasound showed no gallstones or biliary tract dilation. A comprehensive work-up for competing etiologies—including serologies for HAV, HBV, HCV, EBV, HSV1-2, HHV6 and CMV, and autoimmune hepatobiliary antibodies—was negative. Ibrutinib was further reduced to 140 mg daily.

At the end of July 2025, peak values were recorded (ALT 660 U/L, AST 326 U/L, GGT 336 U/L, LDH 398 U/L) with preserved hepatic synthesis and no hyperbilirubinemia, consistent with grade 3 hepatocellular injury. Corticosteroid therapy with prednisone 50 mg/day was initiated while continuing ibrutinib 140 mg/day. Contrast-enhanced abdominal MRI obtained in the same timeframe documented disappearance of most previously described hepatic lesions with only a small residual subcapsular focus in segment VIII, reduction in nodal burden across abdominal stations (including porta hepatis and mesentery), and decrease in spleen size to 13.8 cm. These findings are consistent with a radiologic response of CLL; the biliary tree remained nondilated and portal venous flow was normal.

Liver tests improved rapidly after steroid initiation and dose attenuation of ibrutinib. In early August, AST fell to 99 U/L, ALT to 400 U/L and GGT to 306 U/L, with further decline on subsequent days (AST 59 U/L, ALT 244 U/L, GGT 270 U/L). Venetoclax was commenced at this point without delay (standard ramp-up to target dose), and prednisone was tapered progressively and stopped after 8 weeks.

**Figure 1:**
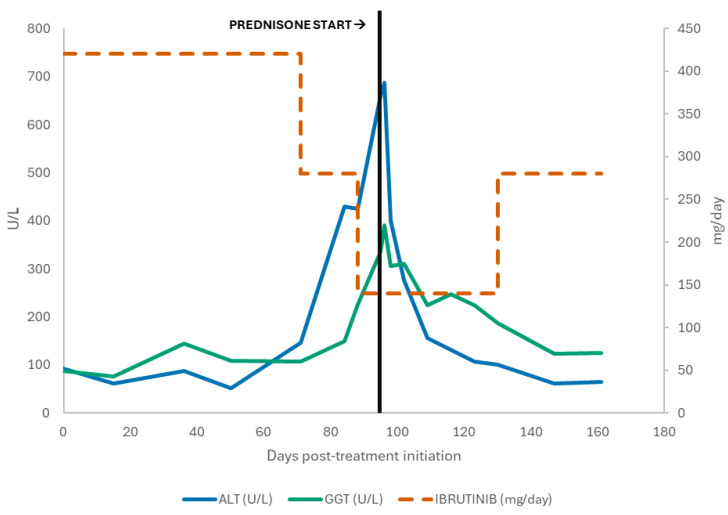
Serum ALT and GGT levels and Ibrutinib dose over time, showing enzyme elevation followed by improvement after Prednisone initiation.

Given continued improvement, ibrutinib was cautiously re-escalated to 280 mg/day in September 2025 without recurrence of hepatocellular injury. The most recent laboratory assessment in late September 2025 showed near-normalization of transaminases (AST 46 U/L, ALT 81 U/L) with persistent but improving cholestatic enzyme elevation (GGT 124 U/L), normal bilirubin (0.9 mg/dL), and normalized leukocyte counts (WBC 5.47 × 10^9^/L; lymphocytes 1.51 × 10^9^/L, Hb 16 g/dL; platelets 158 × 10^9^/L; LDH 240 U/L). At last follow-up (+5 months from starting treatment), the patient remained on venetoclax 400 mg/day plus ibrutinib 280 mg/day in ongoing partial response radiographically and hematologically, and without clinical signs of hepatic decompensation (Figure 1).

Overall, this clinical course is characterized by: CLL with radiologically evident hepatic involvement and baseline steatosis; development of significant hepatocellular enzyme elevations after approximately 10–12 weeks of ibrutinib; exclusion of viral, obstructive, and autoimmune causes; improvement following ibrutinib dose reductions and initiation of corticosteroids; and successful re-challenge at a reduced ibrutinib dose in combination with venetoclax, with sustained biochemical recovery and disease control. These features support suspected ibrutinib-associated liver injury on a background of hepatic steatosis and prior leukemic infiltration, managed with dose modification, immunosuppression.

## 3. Discussion

In the present case, we describe a patient with progressive CLL and radiologically confirmed hepatic involvement who developed acute hepatocellular injury during ibrutinib monotherapy, with subsequent biochemical and radiological improvement following dose reduction and corticosteroid therapy. This report contributes to the limited body of evidence on BTKi-associated hepatotoxicity and underscores the diagnostic and therapeutic challenges in managing liver enzyme elevations in CLL patients with concomitant hepatic infiltration and metabolic liver disease.

### 3.1. Pathophysiological Considerations

The mechanism of ibrutinib-induced hepatotoxicity remains uncertain. Ibrutinib is primarily metabolized by the CYP3A4 isoenzyme and, to a lesser extent, by CYP2D6, yielding several inactive metabolites [15]. Hepatic accumulation of reactive intermediates could provoke direct hepatocellular damage or trigger immune-mediated injury in genetically or metabolically predisposed individuals. Furthermore, ibrutinib’s interference with off-target kinases might alter intracellular signaling cascades relevant to hepatocyte survival or inflammatory modulation [16].

From a mechanistic standpoint, immune-mediated hepatotoxicity remains a leading hypothesis. Ibrutinib modulates several immune pathways, including T-cell receptor signaling and cytokine release [17,18]. Corticosteroid responsiveness, as observed here, supports an immune-inflammatory component to the injury [19].

A further mechanism potentially involved in ibrutinib-induced hepatotoxicity relates to its off-target activity. Although designed to selectively inhibit Bruton’s tyrosine kinase, ibrutinib also binds with high affinity to other kinases expressed in various cell subtypes [20]. This phenomenon is well established clinically: for example, the most frequent adverse event associated with ibrutinib, atrial fibrillation, is attributed to off-target inhibition of cardiac kinases rather than to BTK blockade itself [21]. By analogy, a theoretical mechanism of liver injury may involve the inhibition of TEC kinases expressed in hepatocytes, potentially altering intracellular signaling relevant to cell survival or stress responses [20]. However, this remains speculative, as robust experimental or clinical evidence specifically linking off-target kinase inhibition to hepatotoxicity is not yet available.

The coexistence of leukemic hepatic infiltration could further amplify vulnerability by modifying sinusoidal microcirculation, promoting local inflammation, and disturbing hepatic metabolism.

Another contributory factor in this case may have been pre-existing hepatic steatosis, documented radiologically before treatment initiation. Nonalcoholic fatty liver disease (NAFLD) represents a frequent comorbidity in the general population. Steatosis increases hepatocyte susceptibility to oxidative stress and drug-induced injury by altering mitochondrial function and impairing detoxification pathways [22].

Thus, the observed hepatotoxicity may result from a multifactorial interplay between drug-related immune or metabolic toxicity and an already compromised hepatic background.

### 3.2. Diagnostic Evaluation and Differential Diagnosis

In patients with CLL, the evaluation of liver enzyme abnormalities is inherently complex. Potential etiologies include direct leukemic infiltration, drug-induced liver injury, viral hepatitis reactivation (especially HBV, HCV, HAV, HSV), autoimmune hepatitis, biliary obstruction, ischemic injury, and metabolic dysfunction. In the present case, several elements supported a drug-related hepatocellular injury: (1) a clear temporal association with ibrutinib exposure, typically within 8–12 weeks of initiation; (2) a predominantly hepatocellular pattern of enzyme elevation (ALT > 10 × ULN) with minimal bilirubin increase, excluding cholestatic or mixed patterns; (3) absence of viral markers or autoimmune antibodies; and (4) preserved hepatic synthesis despite marked transaminase elevation.

The radiological re-evaluation during the acute episode provided additional insight. The near-complete regression of previously infiltrative hepatic lesions, in parallel with improvement in lymphadenopathy, excluded disease progression as a cause of liver dysfunction. Similarly, the absence of biliary dilation or vascular compromise ruled out obstructive or ischemic etiologies. Taken together, these findings favored a diagnosis of probable ibrutinib-related DILI. Consistently, the RUCAM score reached a value of 7, supporting a “probable” causal relationship between ibrutinib and the observed hepatocellular injury [23]. A liver biopsy could have clarified the presence of drug-induced hepatitis. However, the procedure was not performed at presentation because the patient exhibited an isolated elevation of transaminases without clinical or laboratory evidence of hepatic insufficiency. In addition, the invasive nature of liver biopsy—and its inherent bleeding risk, potentially accentuated by the antiplatelet effect of ibrutinib—was judged disproportionate in the context of the rapid biochemical improvement observed after initiation of corticosteroid therapy. It was therefore considered appropriate to defer biopsy and reserve it for the event of renewed worsening of liver enzyme levels.

### 3.3. Management Strategy and Rationale

There is currently no standardized approach to managing hepatotoxicity associated with BTKi therapy, largely due to the rarity of reported cases. Clinical judgment must therefore integrate the severity of liver injury, disease control status, and availability of alternative agents. In our patient, stepwise dose reduction of ibrutinib was initially attempted given preserved hepatic function and ongoing CLL response.

However, persistent enzyme elevation prompted the introduction of systemic corticosteroids, which led to rapid biochemical improvement. This suggests that an immune-mediated mechanism could have contributed to the hepatic injury. The use of corticosteroid therapy in our case of ibrutinib-induced hepatic toxicity aligns with previous experiences reported in the management of TKI-induced liver injury, such as that caused by imatinib. Some case series have demonstrated that corticosteroid administration can promote a rapid normalization of liver function tests, allowing the continuation or reintroduction of TKI therapy [24,25]. These findings suggest a key role for steroids in modulating the hepatic inflammatory response triggered by these agents.

The decision to maintain ibrutinib at a reduced dose while introducing venetoclax was based on several considerations. First, complete discontinuation risked rapid disease progression given hepatic infiltration and high disease burden. Second, early reports indicate that partial dose reduction may mitigate toxicity while preserving therapeutic efficacy in sensitive CLL clones. Third, combining venetoclax allowed disease control through a complementary apoptotic pathway (BCL-2 inhibition), reducing dependence on full-dose BTKi activity. The subsequent normalization of transaminases, radiologic improvement, and maintenance of hematologic response without recurrence of liver injury support the safety and effectiveness of this tailored approach.

### 3.4. Comparison with Previously Reported Cases

Four cases of severe ibrutinib-related liver injury have been reported to date, three in patients with Waldenström’s macroglobulinaemia (WM) and one in relapsed/refractory CLL. Nandikolla et al. described the first case of a patient with CLL who developed a predominantly cholestatic DILI two weeks after starting ibrutinib; liver tests improved only partially after drug withdrawal, and the patient ultimately died from progression of the underlying CLL [11]. Kahn et al. reported a 59-year-old woman with refractory WM who developed a hepatocellular pattern of injury nine months after treatment initiation, with liver biopsy showing acute hepatitis with mixed inflammatory features and hepatocellular cholestasis. Biochemical liver tests normalized within 60 days after discontinuation of ibrutinib [12]. Kleijwegt et al. described a 48-year-old woman with WM who developed acute liver failure 11 weeks after exposure to ibrutinib, with marked transaminase elevation, hyperbilirubinaemia and coagulopathy; liver function normalized fully within five weeks after drug withdrawal, and the patient subsequently transitioned to an alternative line of therapy [13]. Finally, Tafesh et al. reported a 77-year-old woman with WM who developed severe mixed hepatocellular–cholestatic DILI eight weeks after starting ibrutinib; the episode was accompanied by impaired hepatic function, with elevation of bilirubin and prolongation of prothrombin time. She was treated with drug discontinuation and high-dose corticosteroids, achieving complete resolution of liver injury within seven weeks [14].

When compared with previously published reports, our case shows both converging features and important distinctions. The timing of onset in our patient—approximately 80 days after starting ibrutinib—falls well within the wide interval observed in the literature, ranging from early presentations at two weeks to delayed onset after several months. Unlike most documented cases, in which liver biopsy was performed, no biopsy was undertaken in our patient. In the previously reported cases, histology consistently demonstrated a characteristic pattern of ibrutinib-related DILI, combining hepatocellular injury with variable cholestasis and mixed inflammatory infiltrates. Management represents the most salient divergence. In all published cases, ibrutinib was permanently discontinued, and in one case systemic corticosteroids were added. In contrast, our patient was treated with corticosteroids while continuing ibrutinib at a reduced dose, making this the only reported case in which BTK inhibition was maintained despite the occurrence of liver injury. Whereas recovery in the literature universally followed drug withdrawal, our patient achieved biochemical resolution without discontinuing ibrutinib, thus preserving disease control. Our early intervention probably prevented the liver failure which would have been a strong indication for drug discontinuation.

Although only a handful of cases have been published, the available reports allow some cautious synthesis. Across the literature, severe ibrutinib-associated hepatotoxicity emerges as an idiosyncratic reaction that generally develops within the first months of therapy, occasionally appearing much later. Management in previously published cases has been largely uniform: ibrutinib was permanently discontinued, and corticosteroids were administered only in the most severe presentations. In all reported patients who recovered, improvement occurred after drug withdrawal, supporting treatment cessation as the most evidence-based approach so far. In this context, our case provides an important addition. Biochemical resolution was achieved while continuing ibrutinib at a reduced dose alongside systemic corticosteroids—an approach not previously documented. Although this single experience cannot redefine standard practice, it suggests that, in carefully selected patients and under close monitoring, continuation of BTK inhibition may be feasible. Further reports will be required to determine whether this strategy can be safely adopted more broadly.

Bruton tyrosine kinase inhibitors of the second generation, such as acalabrutinib and zanubrutinib, have been developed to provide more selective BTK inhibition with reduced off-target kinase activity and an improved safety profile [26,27]. Severe hepatotoxicity related to zanubrutinib has been reported only once: Atallah et al. described a biopsy-proven case of zanubrutinib-induced DILI, with a hepatocellular pattern of injury arising after 30 months of therapy and complete normalization of liver tests following drug withdrawal; Zanubrutinib was permanently discontinued, and the patient subsequently transitioned to an alternative line of therapy [28]. In contrast, to the best of our knowledge, no cases of clinically apparent hepatotoxicity have been reported to date with acalabrutinib or pirtobrutinib [27,29]. These observations suggest that clinically significant liver injury may be less frequent with newer-generation BTK inhibitors, although continued post-marketing surveillance is needed to confirm their long-term hepatic safety.

## 4. Conclusions

This case highlights that Ibrutinib can cause significant hepatic toxicity, warranting careful monitoring of liver function during therapy. Prompt administration of corticosteroids proved effective in controlling liver injury, allowing continuation of treatment at reduced doses. This approach enabled adequate disease control without further complications. The case underscores the importance of individualized and multidisciplinary management when using Bruton’s tyrosine kinase inhibitors.

## Data Availability

The original contributions presented in this study are included in the article. Further inquiries can be directed to the corresponding authors.
